# A new treatment approach – Eplerenone - in central serous chorioretinopathy - Case report

**Published:** 2016

**Authors:** M Cioboata, C Alexandrescu, CA Hopinca, MC Pienaru, A Merticariu, S Schmitzer

**Affiliations:** * Clinical Emergency Eye Hospital, Bucharest, Romania; ** Department of Ophthalmology, University Hospital, Bucharest

**Keywords:** central serous chorioretinopathy, Eplerenone, mineralocorticoid receptors

## Abstract

We report the case of a 46-year-old patient, medical doctor with a relapsing unilateral CRSC-Central Serous Chorioretinopathy who was treated after an initial medical therapy (oral carbonic anhydrase inhibitor, oral antihistamines, non-steroidal anti-inflammatory drugs - systemic and topical), with an oral aldosterone antagonist-Eplerenone (Inspra), resulted in significant anatomic and visual improvements.

**Abbreviations:** CRSC = Central Serous Chorioretinopathy, R.E. = right eye, L.E. = left eye, BCVA = best corrected visual acuity, RPE = retinal pigment epithelium, OCT = optical coherence tomography, FDA = food and drug administration.

Central Serous Chorioretinopathy is a relatively rare cause of visual impairment, which typically affects the adult males (20-50 years old), in whom a serous detachment of the neurosensory retina occurs over an area of leakage from the choriocapillaris through the retinal pigment epithelium (RPE). The other causes for RPE leaks, such as choroidal neovascularization, inflammation or tumors should be ruled out to make the diagnosis [**1**,**2**].

It was reportedly associated with type A personalities, stress, pregnancy, Cushing’s disease and numerous drugs-notably corticosteroids and is common in Caucasians, Asians, and Hispanics [**1**,**3**,**4**] with vision that ranges from 1 to 0.1 , in most patients better than 2/ 3 [**4**].

The overactivation of mineralocorticoid receptor pathway in choroid vessels is presumably involved in the still unknown etiology of CRSC [**5**] 

## History

A 46-year-old male with no considerable familial and personal history was referred to our clinic reporting a sudden loss of visual acuity on L.E., metamorphopsia and a positive central scotoma. After the first presentation, he continued to access for decreased vision after a period of recovering underlying medical treatment.

## Clinical examination

At the first examination, the patient had BCVA R.E.-1, BCVA L.E.-0.4 and IOP: R.E. - 18 mm Hg, L.E. - 15 mm Hg.

**Fig 1:**
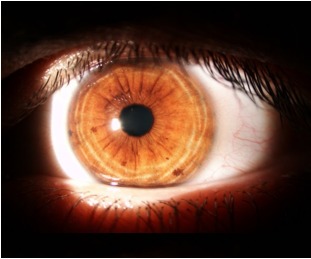
**Fig. 1** Left anterior segment - normal aspect

The biomicroscopy of the anterior pole revealed normal aspects on both eyes (**Fig.1**).

**Fig 2:**
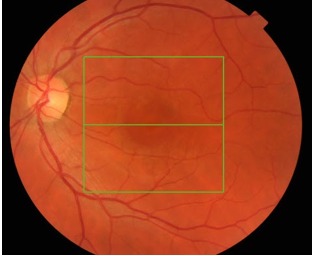
**Fig. 2**L.E. fundus image showing a round area of subfoveal fluid

The ophthalmoscopy of the R.E. did not show any pathological change, but in the L.E., a localized round area of subretinal fluid was remarked (**Fig. 2**).

### Ancillary testing

**Fig 3:**
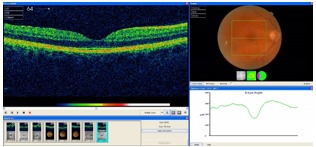
**Fig. 3**Right eye OCT – normal aspect

**Fig 4:**
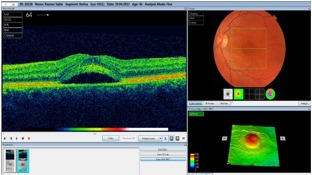
**Fig. 4**Left eye OCT showing serous subfoveal detachment of the neurosensory retina

The R.E. OCT showed no pathological change (**Fig. 3**), but, in the L.E., a serous subfoveal detachment of the neurosensory retina was depicted (**Fig. 4**).

**Fig 5:**
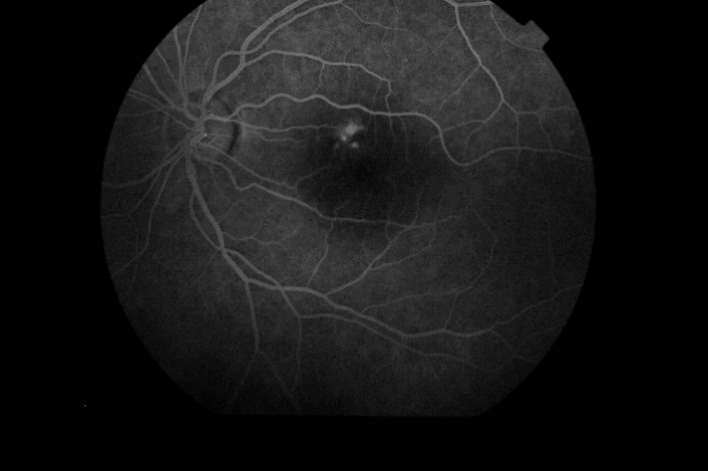
**Fig. 5**Fluorescein angiography: early hyperfluorescent area near the superior temporal vessels with constant size in late phases; two small hyperfluorescent spots with leakage in late phases

The site of fluid effusion could be identified with the aid of fluorescein angiography: early hyperfluorescent area near the superior temporal vessels with a constant size in late phases; two small hyperfluorescent spots with leakage in late phases (**Fig. 5**).

After these clinical and paraclinical exams, the diagnosis of CRSC in L.E. was confirmed and treatment was initiated with nonsteroidal anti-inflammatory drugs (systemic and topical), systemic carbonic anhydrase inhibitor and systemic antihistamines.

At the second month follow-up, the L.E. OCT demonstrated the partial remission of the serous detachment and the patient had a 0.8 acuity in the L.E.

Two months later, the patient presented again with a decreased vision on L.E. (0.5.).

Our patient (also a doctor) did his own research about the available therapy for his condition and asked about the novel treatment with Inspra (Eplerenone – a diuretic).

After this discussion, we decided to initiate the therapy with Inspra. 50 mg was the initial dose once a day, titrated to 25 mg once daily, with monitorization of serum potassium levels and blood pressure level.

At the 3 months follow up treatment, the BVCA was recovered to 0.9 and he maintained this acuity one year after, but when he interrupted the treatment, the disease relapsed.

## Discussion

Eplerenone, a selective aldosterone-receptor antagonist and potassium-sparing diuretic that was originally approved in 2002 by the FDA for treatment of hypertension, was recently shown to improve visual acuity and significantly decrease central macular thickness in a small series of patients with chronic CSCR. The medication is generally well tolerated but drug interactions must be ruled out prior to the initiation and serum potassium and blood pressure must be monitored during treatment. Larger, prospective, placebo-controlled studies are under way to further investigate the efficacy of this treatment option [**5**].

## Conclusion

In our case, the Eplerenone treatment was associated with a significant reduction in subretinal fluid level and an improvement in visual acuity, but only dose dependent. The discontinuation of the treatment induced a relapse of the disease.
